# Medicine

**Published:** 1910-12

**Authors:** George Parker


### Progress of the Medical Sciences.

### MEDICINE.

**Infantile Paralysis.**—The remarkable increase in the outbreaks of infantile paralysis during the last few years has led to many investigations and a better knowledge of the subject. In the sporadic form it has long existed in most countries, but of late at least seventy epidemics have appeared, a great number of which were in Scandinavia and America, while only two or three small ones have been recorded in this country. In the United States since 1907 a series of serious outbreaks have spread from sea to sea. In 1909 there were 569 deaths reported, and if we reckon the mortality at 12 per cent., there must have been nearly 5,000 persons attacked, of whom a large proportion were crippled for life. McClanahan[1] reported 1,000 cases in one district twenty miles by twenty-four. As usual the season was chiefly July and August. The fatal cases here numbered 137, and were generally due to the respiratory centre being involved. A large number of patients in this particular outbreak recovered without lasting paralysis.

Simon Flexner shows that before reaching America the epidemic form had spread over a large part of Europe, and he thinks it possible that the disease was carried over by Scandinavian immigrants. In America it spread along the chief lines of communication, and behaved much as any other mildly infectious disease. He finds evidence that the disease can be carried by a healthy person after contact with a patient, and that the incubation period is about nine days, though with very wide variations, a time which is ample for the Atlantic voyage.

W. W. Treves[2] has described fully an outbreak of eight cases in England during 1908, and mentions two other small ones. In Bristol some forty cases occurred in 1909, an account of which is in course of publication. In Vienna and Lower Austria 266 cases were seen in 1908, and in Arnsberg 333. In Melbourne Stephens met with 137 cases, with six deaths only, in 1908. An outbreak recorded by Calverly as far back as 1894 showed 136 cases in human beings, and others in cattle and fowls. No pathological examinations were made, and it is possible that this was a distinct disease, but a curious mortality in fowls has been noted in several epidemics.

Treves discusses Wickham's analysis of four Scandinavian epidemics, one of which comprised 1,035 cases. The latter claims to have shown the infectiousness of the disease from instances

[1] *J. Am. M. Ass.*, 1910, lv. 1160.
[2] *Brain*, 1909, xxxii. 283.

where it broke out in a healthy place immediately after the arrival of individuals from an infected one, and in other cases by direct contact. He was thus able to trace it from village to village, and it has since been made compulsorily notifiable in Norway. However, this is no more than occurs with pneumonia, which occasionally takes an epidemic form. There are, as we shall see, other differences in the behaviour of the disease when epidemic, but in typical cases it does not seem that any real distinction can be drawn between the sporadic and epidemic types, either pathologically or clinically, though the propagation of the organism concerned has apparently only been carried out from epidemic cases.

Wickham[1] records the concurrence, among the normal cases, of others showing ascending myelitis, bulbar and pontine inflammation, encephalitis, meningitis, ataxia, polyneuritis, paralysis without febrile or gastro-intestinal symptoms, and abortive forms without paralysis. He found a mortality in 1905 of 11 per cent. in patients under ten, and about 26 per cent. in older persons. In another epidemic we meet with a mortality of 33 per cent. Holt and Bartlett[2] conclude that in a number of epidemics the mortality was 12.1 per cent. They explain this high rate, as compared with that of the sporadic form, by saying that the fatal bulbar cases are not recognised as infantile palsy when met with sporadically. Gowers,[3] in referring to these differences, notes also the number of complete recoveries in epidemics. Thus in Boston out of 628 cases there were 10 per cent. without lasting paralysis. Another point is the number of older patients, and in these the initial symptoms tend to be more severe. It has been commonly held that there are two forms of attack: (1) the common febrile form, lasting some days, with pains in the back and limbs; and (2) a sudden paralysis during perfect health or following a shock or chill. Krause,[4] however, in the Westphalian outbreak, 436 cases met with gastro-intestinal symptoms in 90 per cent. of the cases, and swelling of the intestinal glands, while in certain families some individuals had paralysis and others diarrhœa only. In Hessian and some American cases a great reduction of leucocytes was noticed, but it is not known whether this is universal.

As to the pathology, much light has been thrown on the subject by Flexner and Lewis, Römer, Llandsteiner and Levaditi. They have succeeded in propagating the disease in monkeys for twenty-five generations with the usual paralytic symptoms. Geirwold's diplococcus does not seem to be the causal

1 *Ztschr. f. klin. Med.*, lxii., 1907.
2 *Am. J. M. Sc.*, 1908, cxxxv. 647.
3 *Brit. M. J.*, 1910, i. 305.
4 W. H. Wynn, *Birmingh. M. Rev.*, 1910, lxvii. 142, and *Deutsche Med. Wchnschr.*, 1909, Oct. 21st.

agent. The real organism is one of the most minute known, and can pass through the finest porcelain filters. An emulsion of an infected spinal cord remains virulent for weeks, and is unaffected by freezing or drying or by the addition of glycerine, though the addition of very weak disinfectants or heating to a temperature of 110° soon proves fatal to its activity. Injections of the spinal fluid do not convey the disease, but an emulsion of the salivary glands or of the cord does.[1] Injections may be made subcutaneously, intraperitoneally, or with more certainty into the brain or under the nasopharyngeal membrane. The last Flexner[2] thinks is the usual route of infection, and the source whence it is scattered about.

The spinal fluid in monkeys, and probably in man, in the earlier stage before the onset of paralysis is opalescent, coagulable, and shows an excess of cells; afterwards it is clear, does not clot, but shows some excess of lymphocytes, differing in this way from that of epidemic meningitis. An attack in man gives immunity for an unknown period, but at present no attenuated virus has been found capable of preventing the disease. Lumbar injections of serum from immunised animals or men, given early, seem to postpone or sometimes to prevent paralysis, and possibly an effective serum may be eventually obtained from the sheep. However, the subject is in the experimental stage, and no reliable remedy can be offered at present. Urotropin has been recommended on theoretical grounds. Formaldehyde seems effective as a disinfectant and for inhalations. No animals except monkeys have been successfully infected by human poliomyelitis, though several species of animals suffer from a similar disease.

A research is now being made as to whether the nasal secretions convey the disease, whether there are intermediate hosts, what is the period of infectiousness, and how a diagnosis can be made before paralysis comes on. Williamson thinks that the loss of the Achilles reflex is a useful distinction from other forms of paralysis, and notes that the "vibrating sensation" is not lost, as it may be in peripheral neuritis.

F. E. Batten[3] discusses two cases in which the disease seems to have been acquired during intrauterine life. Both pathologically and clinically they were typical cases.

Since in epidemics cases are seen where the brain only is affected, or no paralysis at all occurs, the name poliomyelitis becomes inappropriate, and this leads to the question whether certain groups of cerebral affections which occur sporadically are not after all forms of infantile palsy. Richard Miller[4] places under this head (1) encephalitis of the cortex (Strumpell's

[1] Williamson, *Med. Chron.*, 1910, lii. 32.
[2] *J. Am. M. Ass.*, 1910, lv. 1105.
[3] *Brain*, 1910, xxxiii. 149
[4] *Practitioner*, 1910, lxxxv. 96.

hemiplegia of children); (2) certain affections of the cranial nerves and the bulb; (3) Batten's acute ataxia with hypotonus; (4) Gordon Holme's acute tremor with hypertonus; and (5) thalamic encephalitis with athetosis. He argues that these are usually due to infantile palsy, attacking the brain instead of the cord. Leonard Parsons[1] supports this view in a most interesting paper, and gives two cases in which typical lesions of infantile paralysis followed attacks of tremor of the Gordon Holmes type. If these various theories are proved, our conception of infantile paralysis will be much extended. It will include both sporadic and epidemic cases, and the symptoms will vary with the part of the nervous system which happens to be attacked.

**Radioscopy of the stomach** is of great value in diagnosing dilatation, new growths, pyloric stenosis and the hour-glass condition. Some serious poisoning cases have arisen from the use in this method of bismuth nitrate in large doses. A. W. Crane[2] still recommends it, but follows it up with a full dose of Epsom salts. Gray[3] Hürter and Jordan use the carbonate in doses of fifteen grains up to three or four ounces. Hertz[4] employs an ounce or more of the oxychloride. Thus Hürter gives a capsule holding fifteen grains to show the lower margin of the stomach, then half an ounce in a wineglass of water to show the contour, with a seidlitz powder to show up the fundus, or a meal of porridge mixed with bismuth may be used. Crane gets good results from photos, but others prefer the screen. Lange[5] showed an excellent sketch from the picture of two cases of carcinoma, where the affected area was a blank translucent space with irregular edges. Hürter[6] finds that in malignant disease the shadow is narrow, and the pylorus is not carried far to the right, all of which is reversed in benign affections. Again, on pressing the abdomen with the finger-tip a large, irregular, clear space appears. In the normal person this spot is very small, and in simple inflammation large but regular. Hertz and several others hold that in health the lowest part of the greater curvature usually reaches below the umbilicus when the patient is upright. The body of the stomach is then nearly vertical, and the dome-like fundus lies above the cardiac orifice and contains gas. The pyloric vestibule turns upwards and backwards at less than a right angle to the cardiac part. The shape of the whole organ is very much that of a long stocking, and Hertz shows that when empty

[1] *Birmingh. M. Rev.*, 1910, lxvii. 148.
[2] *J. Am. M. Ass.*, 1909, liii. 1962.
[3] *Lancet*, 1908, ii. 224.
[4] *Quart. J. Med.*, 1910, iii. 373.
[5] *J. Am. M. Ass.*, 1910, liv. 873.
[6] *Arch. f. Verdauungskr.* 1910, xvi. 202.

the lower two-thirds contract to form a rod or tube without a lumen, so that it resembles a pear with a very long, curved stalk. This stalk dilates as food descends, and when the latter reaches the middle of the stomach peristaltic waves commence to drive it forwards. Carbohydrates leave the stomach quicker than proteids, and these sooner than fats. Grützner argued that later portions of food remain in the centre of the mass undergoing salivary digestion, while the first portions eaten are acidified and pass on; but Hertz shows that in man they tend to mix quickly. Still, in taking out a test meal it is important to remove the whole contents, and not to estimate the HCl. from one part only. Water and egg albumen leave an empty stomach rapidly without causing any flow of HCl. Hence they are often retained by patients when even milk is vomited. The increased peristalsis in obstruction is easily seen, and the position and character of œsophageal strictures.

George Parker.

